# Myxoid mesenchymal neoplasm presenting as massive arm and chest wall oedema with pleural effusion

**DOI:** 10.3332/ecancer.2015.590

**Published:** 2015-11-05

**Authors:** Vidya B Pai, Rahul Ravilla, Matthew Lindberg, Matthew Steliga, Konstantinos Arnaoutakis

**Affiliations:** 1 Department of Internal Medicine, Paediatrics, University of Arkansas For Medical Sciences, AR 72205, USA; 2 Department of Internal Medicine, University of Arkansas for Medical Sciences, AR 72205, USA; 3 Department of Pathology, University of Arkansas For Medical Sciences, AR 72205, USA; 4 Division of Thoracic Surgery, University of Arkansas For Medical Sciences, AR 72205, USA; 5 Division of Haematology–Oncology, University of Arkansas For Medical Sciences, AR 72205, USA

**Keywords:** extracellular matrix, malignant pleural effusion, neoplasms, sarcoma

## Abstract

Myxoid mesenchymal tumours are a heterogeneous group of neoplasms characterised histologically by their abundant mucoid and myxoid extracellular matrix (ECM). Encompassing a broad spectrum of clinical behaviour ranging from benign to malignant, there are more than 60 reactive and neoplastic entities currently classified under its domain. Its varied clinical and histopathologic features continue to pose a diagnostic challenge to clinicians and pathologists. Here, we describe a rare case of myxoid mesenchymal tumour presenting as oedema of the upper extremity with pleural metastasis and partial response to chemotherapy, which to the best of our knowledge has not yet been described in the literature.

## Clinical case

**Figure 1.**
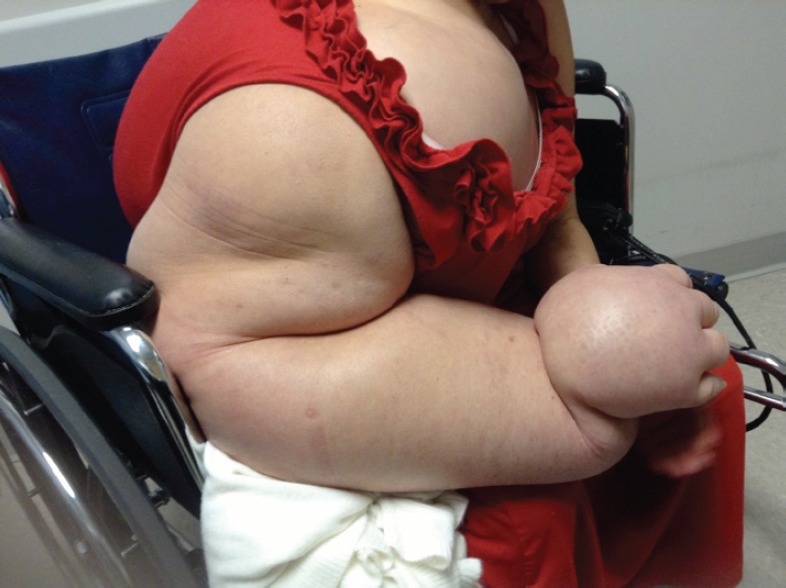
Initial presentation with massive swelling of right hand with involvement of hemithorax.

**Figure 2.**
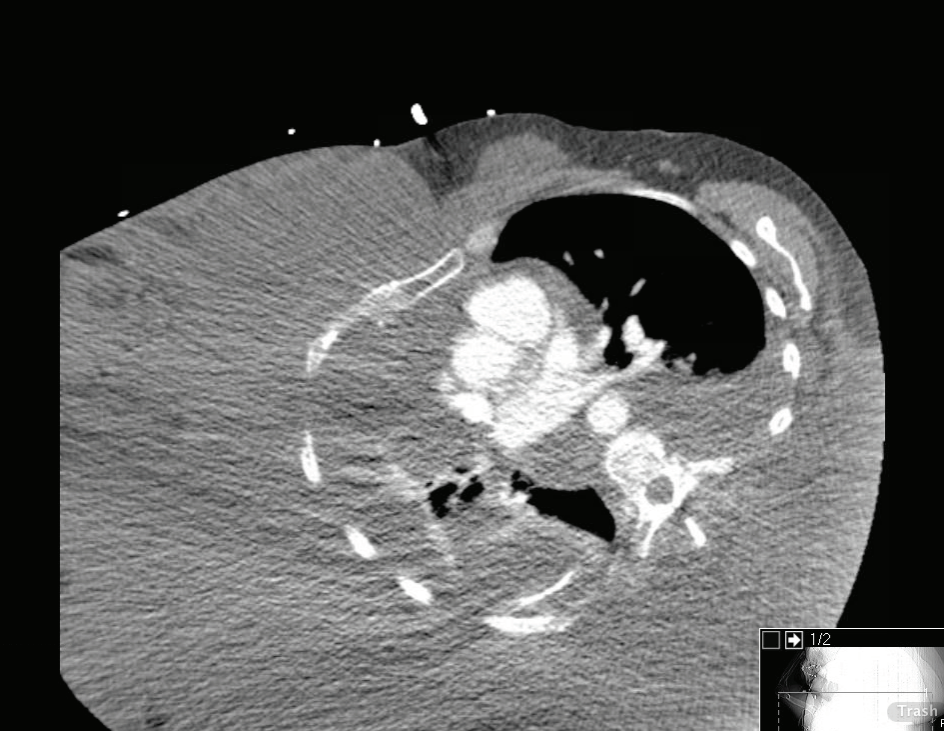
Axial CT chest demonstrating swelling of right hemithorax.

**Figure 3.**
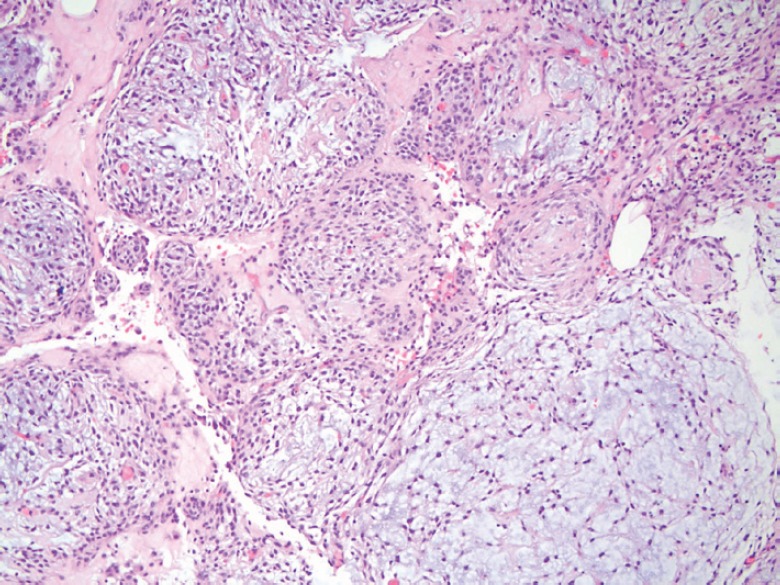
Pathology at 10× magnification showing a nodular, neoplastic proliferation composed of small ovoid to spindled cells demonstrating relatively bland, monomorphic nuclei and located within a variable fibrous to myxoid stroma.

**Figure 4.**
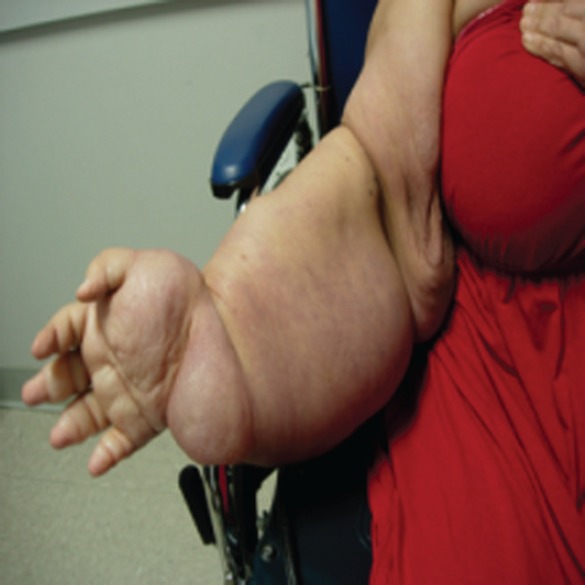
Improvement in swelling after 1 cycle of chemotherapy.

A 49-year-old Hispanic woman presented to the ambulatory medicine service for worsening oedema of her right upper extremity. Six years prior to presentation, she noticed painless swelling of her right thumb, which she initially attributed to an injury that occurred while working in a chicken-processing plant. She reported receiving repeated local injections of an unknown antibiotic in Honduras over the course of 2 weeks without relief. As time progressed, so did the swelling with extension from her hand to shoulder with eventual involvement of her right chest wall and hemithorax (Figure 1). Her medical evaluation at that time was extremely limited and included a complete blood count and an X-ray, both of which she reported were normal, and hence, no further workup was sought. Eighteen months before presentation to the clinic, she developed dyspnoea on exertion with occasional cough. She reported right arm and back pain from the significant swelling with associated right chest wall discomfort. Apart from postprandial nausea, she denied any fever, night sweats, weight loss, or lower extremity oedema. A computed tomography (CT) scan of the chest with contrast revealed soft tissue oedema without evidence of thrombus or obstruction in the venous system (Figure 2). Further evaluation for multiple infectious aetiologies, including tularaemia, coccidiodes, and malaria, were negative; however, she did have a positive purified protein derivative (PPD) test for which she received and completed latent tuberculosis treatment. Subsequent lymphoscintigraphy showed evidence of lymphatic obstruction at the level of the distal forearm and she was referred to a lymphedema clinic for compressive therapy. Her oedema continued to progress resulting in a large right-sided exudative chylus pleural effusion requiring thoracentesis. Fluid analysis for at that time was negative for malignancy and cultures were negative for acid-fast bacilli. She again presented two months later with dyspnoea and was found to have a re-accumulation of her pleural effusion at which time she underwent pleuroscopy with biopsy and pleurodesis with pleural drain placement. Although repeat pleural fluid analysis was negative for malignancy, thoracoscopy revealed a pleural space that was densely adherent in multiple locations with gelatinous, firm material. Pathological examination showed sections of very nodular neoplastic proliferation of small spindled to ovoid cells with eosinophilic cytoplasm and bland nuclei within a predominantly myxoid stroma. A ‘whorled’ cellular morphology was identified multifocally without mitotic figures or necrosis. Immunohistochemically, the tumour cells involved were positive for epithelial membrane antigen and progesterone receptor but negative for Ewing sarcoma break point 1 gene translocation, smooth muscle antigen, cytokeratin AE1/AE3, S100, calretinin, microphthalmia-associated transcription factor (MITF), and claudin 1 (Figure 3). The cytogenetics from the cell cultures of the pleural biopsy revealed complex chromosome translocations and deletions 45,XX,-6,inv(9)(p24q32)x2[14]/90,idemx2[3]/44,idem,dic(19;19)(q13.4;q13.4)[1]/90,idemx2,-6,+7,+11,dic(19;19)[1]/46,XX[1]. Surveillance magnetic resonance imaging (MRI) brain showed no intracranial or meningeal masses. Tissue specimens were referred to a sarcoma expert who identified it as an unusual variant of myxoid sarcoma that could not be classified. Chemotherapy was initiated with doxorubicin that she received every 21 days for 4 cycles. After one cycle of chemotherapy, she had a drastic improvement in her symptoms and physical appearance (Figure 4). Due to her poor performance status in addition to side effects from her prior regimen, her chemotherapy regimen was changed to gemcitabine. Unfortunately she continued to decline and ultimately opted for hospice care.

## Discussion

Sarcomas are a group of rare neoplasm arising from mesenchymal origin that represent 1% of all adult malignancies and 12% of paediatric cancers [1]. It can be divided into two major categories: soft tissue sarcomas (STS) and bone sarcomas that represent 80% and 20%, respectively, the former of which we will focus on here. Of particular interest are myxoid tumours, a complex group of mesenchymal neoplasms identified by the presence of large amounts of extracellular myxoid matrix. The term myxoid describes the gelatinous mucopolysaccharide-rich water content that is produced by tumour and which does not exist in normal human tissue [2]. The myxoid tumours are classified based on the origin of tumour cells as follows: myxofibrosarcoma from fibroblastic origin, myxoliposarcoma from adipose tissue and tumours of uncertain differentiation such as acral myxoma and intramuscular myxoma. They are further classified based on their malignant potential into benign, locally aggressive and malignant tumours [3]. The clinical presentation of sarcomas varies depending on the origin of the primary tumour. While soft tissue sarcomas can occur anywhere in the body, the majorities arise from the buttocks and groin (46%), torso (18%) upper extremities (13%), retroperitoneum (13%) and less frequently the head and neck region (9%) [4]. In the case of myxoid sarcomas, the presentation is quite variable. Patients often present with a gradually enlarging painless mass although some may experience symptoms associated with local mass compression, including paresthesia and localised oedema. Benign tumours, such as superficial angiomyxoma, acral myxoma, and intramuscular myxoma, are localised with well-demarcated borders, while malignant tumours, such as myxofibrosarcoma, are mostly superficial without distinct borders and tend to spread along the fascial planes frequently leading to incomplete surgical resection and a high recurrence rate [2]. Like other sarcomas, the tendency for pulmonary metastasis is high in this subtype. The exception to this is myxoliposarcoma that has higher predisposition to extra-pulmonary metastasis especially osseous metastasis to spine [5]. The gold standard for the diagnosis of myxoid tumours is histopathological examination; however, this often poses significant challenges due to the high myxoid content and the fact that most tumours are either hypo- or paucicellular. In such cases, cytogenetic studies assist in diagnosing tumours with characteristic translocations such as myxoid liposarcoma [*t*(12:16)(q13;p11)], myxoid chondrosarcoma [*t*(9;22), *t*(9,17)] [3]. In most cases, the cytogenetic studies are usually complex like in our case. Furthermore, the immunohistochemistry of stromal cells can aide in diagnosis like MUC4 stain in low-grade fibromyxoid sarcoma. The high water content of the myxoid matrix leads to hyper-intense T2 diffusion-weighted signals on MRI compared to the surrounding tissue that helps in identifying patterns and borders of the tumour. In aggressive angiomyxoma, there is classic swirling or layered pattern, while myxoliposarcomas shows multilobulation with septation. Myxofibrosarcoma is identified by its classic ‘tail sign’ concurring with infiltrative spreading nature [6, 7].

## Prognosis/Treatment

A number of prognostic factors for STS survival exist and include patient age, tumour size, stage, grade, subtype, and margin of resection [8]. Depending on these prognostic factors, a multidisciplinary approach to treatment should be sought include surgery, chemotherapy, and radiation therapy. Nevertheless, wide surgical resection with tumour-negative margins (R0 resection) remains the cornerstone of treatment for locally advanced disease and may show increased overall survival when combined with radiation therapy. Furthermore, chemotherapy may be considered for palliation. It is well known that the standard chemotherapeutic agents for soft tissue sarcoma are anthracyclines with doxorubicin showing the best established single agent response rate ranging from 10–25% [9]. A landmark trial by the European Organisation for Research and Treatment of Cancer (EORTC) showed that when in used in combination, doxorubicin and ifosfamide showed a significant progression free survival rate when compared to single-agent doxorubicin, although overall survival benefit remained the same [10]. In patients with poor performance status such as ours, gemcitabine may be used as an alternative and has shown modest activity in Phase-II clinical trials [11]. A randomised double-blind Phase-III trial in 2013 (PALETTE) showed pazopanib, a targeted tyrosine kinase inhibitor indicated for the treatment of non-resectable or metastatic non-adipocytic soft tissue sarcomas in patients who have previously received standard anthracyclines chemotherapy yielded a median progression-free survival of 4.6 months compared to 1.6 months for placebo with an increase in overall survival [12]. One drug that showed promise in cases of advanced translocation related sarcomas is trabectedin. Acting on DNA repair mechanism, it has been shown to improve progression-free survival in patients who already received anthracycline-based therapy [13]. Although the study population is comprised of predominantly leiomyosarcoma and liposarcoma, the drug is being studied in other sarcomas as second-line monotherapy or in combination with anthracyclines. It is currently approved in Europe and received orphan drug status from FDA to treat advanced sarcomas.

## Conclusion

In conclusion, early definitive diagnosis is crucial to determining appropriate treatment strategies. In the case of our patient, her swelling was likely due to lymphatic obstruction from the slow progressive growth of the myxoid neoplasm. A diagnosis established earlier in her disease process may have led to potential surgical resection; however, given her prolonged clinical course and late presentation with massive involvement of the entire right arm and hemithorax, systemic chemotherapy and supportive care were the only treatment options.

## Conflicts of interest

The authors declare that they have no conflicts of interest.
